# A five-primary Maxwellian-view display for independent control of melanopsin, rhodopsin, and three-cone opsins on a fine spatial scale

**DOI:** 10.1167/jov.22.12.20

**Published:** 2022-11-29

**Authors:** Thomas W. Nugent, Andrew J. Zele

**Affiliations:** 1 Center for Vision and Eye Research, Queensland University of Technology (QUT), Brisbane, QLD, Australia

**Keywords:** melanopsin, ipRGC, rods, cones, silent substitution, five-primary display

## Abstract

Independent spatiotemporal control of the stimulation of the five photoreceptor classes requires a display with as many primary lights to probe their isolated spatial and temporal responses. No such system exists with suitable performance properties. We present a system to construct a five-primary display from commercially available three-primary digital light processing projectors. It optimizes the set of five primary lights required to maximize the achievable contrast of a single photoreceptor class in a silent substitution protocol, including where the background chromaticity is first specified. From these chosen five primaries, we describe a method to convert complex three-primary (RGB) images to five-primary representations with complete specification of the photoreceptor excitations at each pixel. Key to enabling this multiple display system with a single HDMI connection is a novel control protocol implemented in a deterministic field-programmable gate array controller that splits the data into five video streams to allow nearly synchronous presentation of primary image data through multiple displays. Each pixel is controlled over 9.5 bits for each primary over a single frame for measurement of threshold-level vision. In addition to a large contrast gamut, the Maxwellian view offers high retinal illumination to support the investigation of five opsin-based responses to complex spatiotemporal images with a truly silent substitution protocol, while avoiding the confounding effects of uncontrolled photoreceptor excitations as occurs in four-primary systems. The customizable primaries facilitate this display translation to species with different photoreceptor spectral responses, and the optics are designed for integration into microscopes for use as a stimulus generator in physiological experiments.

## Introduction

Human visual sensitivity to light is dependent on the relative activity of five photoreceptor classes and their postreceptoral pathways. The duplicity theory of vision (Schultze, 1867; Kries, 1896; Müller, 1930) had postulated separate and independent functions of the cones during daytime and rods at nighttime. The physiological reality is, however, more complex. Shared neural pathways lead to the dual processing and interaction of rod and cone signals (Polyak, 1941; Daw et al., 1990; Wässle et al., 1995; Sharpe & Stockman, 1999) that modify our visual experience (Buck, 2003; Barbur & Stockman, 2010; Zele & Cao, 2015). Rods can drive visual responses well into the photopic range (Shapiro, 2002; Sharpe et al., 1989; Kremers et al., 2009; Uprety et al., 2022), while melanopsin inputs to the pupil control pathway (Barrionuevo et al., 2014; Zele et al., 2019a) and vision (Zele et al., 2019b, 2020) are first evident in mesopic lighting. The intrinsically photosensitive retinal ganglion cells (ipRGCs) mediate both the intrinsic melanopsin photoresponse and extrinsic inputs from outer retinal rod and cone photoreceptors (Dacey et al., 2005; Nasir-Ahmad et al., 2019; Ostrin et al., 2018) with intraretinal pathways supporting the melanopsin modulation of the cone and rod signals (for review: Grünert & Martin, 2021). An accumulation of evidence now points to melanopsin expressing ipRGCs supporting a diverse range of visual and nonvisual functions, independent of and working in combination with the classical photoreceptor pathways. Melanopsin independently contributes to color perception (Zaidi et al., 2007; Spitschan et al., 2017; Cao et al., 2018; Zele, Feigl, et al., 2018; Zele et al., 2019b; Barrionuevo, Filgueira, et al., 2022), contrast sensitivity (Zele, Feigl, et al., 2018), temporal (Zele, Feigl, et al., 2018) and spatial vision (Allen et al., 2019), adaptation (Pant et al., 2021), and simple reaction times (Gnyawali et al., 2022) and can combine and interact with the rod-mediated and cone-mediated functions to affect brightness perception (Brown et al., 2012; Besenecker & Bullough, 2017; Zele, Adhikari, et al., 2018; Yamakawa et al., 2019; DeLawyer et al., 2020), photophobia (Zele et al., 2020), decision-making (Gnyawali et al., 2022), the perception of time (Yang et al., 2018), contrast discrimination (Zele et al., 2019b), and temporal processing (Uprety et al., 2022). All three photoreceptor classes input to the circadian and pupil control centers, with the ipRGCs forming the principal afferent pupil pathway (Gamlin et al., 2007; Ostrin et al., 2018), the retinohypothalamic tract (Gooley et al., 2001), and for mediating the effects of light on vision, sleep, mood, and arousal (for review: Lucas et al., 2020; Markwell et al., 2010; Feigl et al., 2022; Feigl & Zele, 2014; Houser et al., 2021). Central to these findings were the development of alternative methodological approaches for separating melanopsin from cone (and, sometimes, rod) function.

Four-primary systems have been designed for silent substitution to control mesopic rod–cone interactions using either spatially homogeneous (Pokorny et al., 2004; Sun et al., 2001; Tsujimura et al., 2010) or dynamic spatial stimuli (Bayer et al., 2015). The development of the five-primary photostimulator (Cao et al., 2015) first met the requirement for fine temporal control and large contrast gamuts for independently stimulating all five photopigments across a uniform stimulus field. Spatial control of the melanopsin pathway has been attempted by overlaying the images of two vertically separated liquid-crystal display (LCD) projectors and forming four primaries with interference filters (Allen et al., 2018; Yang et al., 2018; Allen et al., 2019), but four primary projectors leave rhodopsin excitation uncontrolled and rely on the assumption that there exists no rod intrusion under the measurement conditions. Because as little as 3% rod contrast in a melanopsin-directed photopic stimulus can affect the measured visual contrast sensitivity (Uprety et al., 2022), it is necessary to have an instrument capable of spatiotemporal control of the excitations of all five photoreceptor classes. There are systems with four or more primaries for generating melanopsin-directed spatial stimuli. A six-primary system has been developed that uses two digital light processing (DLP) projectors as a backlight for an LCD screen (Hexley et al., 2020); the filtering of the LCD screen dominates the spectral shape of the six primaries in this system, and as a result of the spectral correlation of the primaries, this system must also leave rhodopsin excitation uncontrolled when generating melanopsin-directed stimuli. Another approach (Lee & Ripamonti, 2022) uses a DLP projector that is backlit by a spatially homogeneous five-primary system similar in principle to that developed by Cao et al. (2015); although the DLP projector can switch states at 6000 Hz, it must construct spatial stimuli by progressively adding binary image structures. This approach means that any spatial complexity in the stimuli must be compensated for with lower pixel-level bit depth and effective frame rate of the system. Smoothly changing stimuli (e.g., a spatial sinusoid) will require many hundreds of binary image structures to construct and will result in a frame rate below the temporal integration time of the eye. A six-primary system has also been developed using two DLP projectors where the two sets of native red-green-blue (RGB) primaries have been spectrally filtered with high- and low-pass filters (Yamaguchi et al., 2008; Yamakawa et al., 2019); each projector is controlled by an independent PC for silent substitution (Yamakawa et al., 2019), which will have temporal misalignment between primary image planes without dedicated hardware to align each video output.

Here we present the theory, design, validation, and performance characteristics of a five-primary spatial display that achieves true silent substitution across all five photopigments at 230,400 spatially separate locations within a configurable field of view. The information provided allows researchers to build their own system to their required specifications. The primaries were systematically chosen to maximize the contrast gamut within a five-dimensional photoreceptor excitation space. With 9.5-bit contrast control per primary, this display system can probe threshold-level vision for cone-directed, rhodopsin-directed, and melanopsin-directed stimuli and in the evaluation of interactions between the responses of five photoreceptors in the human eye. Spatiotemporal control of the excitations of the five photoreceptor classes will enable the investigation of the visual functions of ipRGCs while eliminating potential rod and/or cone intrusions and to determine how interactions between the five photoreceptor classes set human visual contrast sensitivity.

## Method

**Figure 1.**
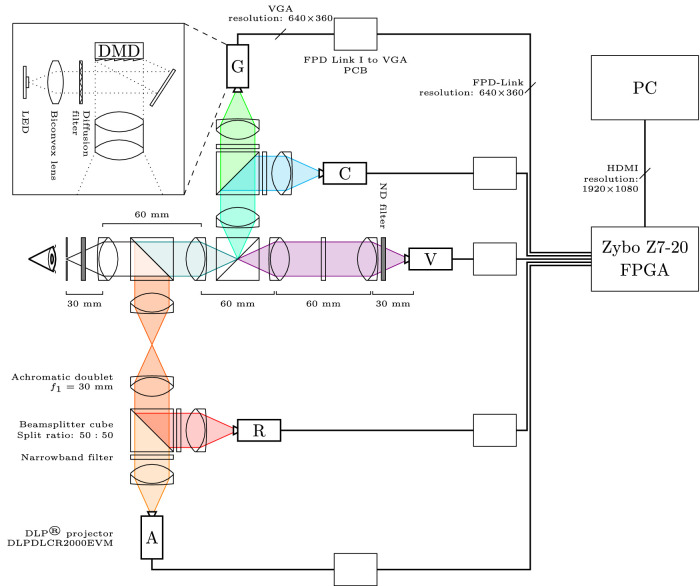
Schematic design of the five-primary display. The optical components merge each of the images of the five displays onto a single axis of projection that is presented in the plane of the pupil in Maxwellian view. The final retinal image size is configurable. The five DLP projectors are controlled via a DLPC2607 controller chip and a custom-designed RGB to FPD Link I PCB. The FPGA takes a high-resolution image and creates five independent image planes for each primary (V = violet; C = cyan; G = green; A = amber; R = red). An optical engine with the custom LED primary is shown in the inset. A side profile of a DLP mounting and alignment stage is shown in Figure 2 and Figure 8.

The five-primary display (Figure 1) is designed for Maxwellian view (Maxwell & Stokes, 1860; Westheimer, 1966; Barrionuevo, Preciado, et al., 2022). Briefly, each primary is projected from one of five DLP Lightcrafter 2000 evaluation modules (EVMs), where each DLP projector has been modified so that it is backlit with a narrowband light-emitting diode (LED). These narrowband LED and interference filter combinations are chosen to maximize the system’s gamut of photoreceptor excitations. The five primary images are merged onto the same axis of projection with a sequence of achromatic doublets and beamsplitter cubes. A central field-programmable gate array (FPGA) (Zybo Z7-20; Xilinx, San Jose, CA) synchronously feeds the projectors image frames by partitioning a higher-resolution (1920 × 1080 pixel), high-definition multimedia interface (HDMI) video input into five lower-resolution (360 × 640 pixel) FPD Link I video outputs. The following details the design and evaluation of the system components.

**Figure 2.**
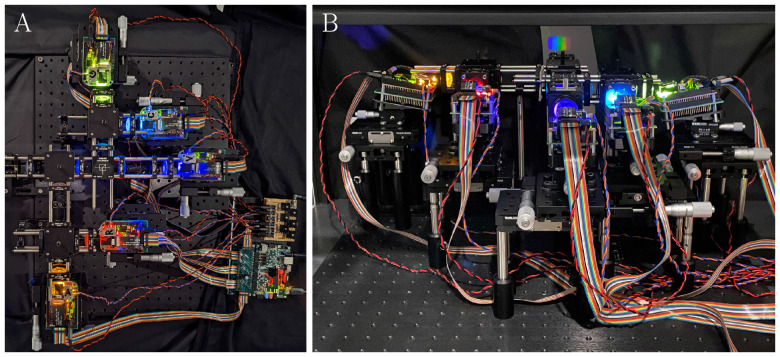
The five-primary display system. (A) Top-down view of the optical system to match the orientation of the schematic in Figure 1. Each DLP is connected via a ribbon cable to the custom-designed driver PCB and Zybo Z7-20 FPGA (lower right side, green PCB). The DLP and LED power is supplied through a custom-designed power supply board (upper right side, brown PCB). The artificial pupil for Maxwellian view is at the left of the middle optical axis. (B) Side view of the optical system with an image of the output of the exit pupil projected onto a screen in Newtonian view. The five primaries (violet, cyan, green, amber, red) are displayed as vertical stripes horizontally separated on the white screen. The system was photographed using a smartphone under dim mesopic lighting to accentuate the light path of each projector. The smaller color gamut of the camera does not accurately render the true color appearance of the primaries.

### Projector control

Each of the five primary images is projected by a Lightcrafter 2000 EVM, which has been modified to homogeneously project a single narrowband LED onto the digital micromirror device (DMD). The EVM is a prebuilt projection system containing the Lightcrafter 2000 chipset family and a Young Optics optical engine. Projected images are generated by the DLP2000 DMD, which consists of a rectangular array of 360×640 micromirrors. Each micromirror is electrically controlled into either an ON/OFF state with an input HIGH/LOW voltage. In an ON state, the DMD reflects the illuminant to the aperture of the projection lens; in an OFF state, the DMD reflects the illuminant away from the aperture of the projection lens and into the nonreflective walls of the optical engine (Hornbeck, 1998; Lee, 2018; Texas Instruments, 2019). The bistable digital micromirror only provides digital control of a pixel state, and so the DMD uses pulse width modulation (PWM) to achieve up to 256 levels for each of the three stock RGB primaries, and with the modification described in this section, we extend this to 768 levels for each primary over a 60 Hz video frame.

We control the DMD with the DLPC2607 controller chip on board the EVM. This chipset inputs 24 parallel data bits formatted as RGB888 video graphics array (VGA) data and outputs Texas Instrument’s proprietary bit plane data format to the DLP2000 DMD. The DLPC2607 controller chip can be configured through interintegrated circuit (I2C) in—out pins, and these settings are configured in the five-primary display to reduce digital image processing in the DLPC2607. On initialization of the system, the Zybo Z7-20 FPGA transmits the appropriate I2C terminal commands to the DLPC2607 chipset.

Control of the RGB888 input into a single Lightcrafter 2000 EVM requires a 28-signal parallel bus. The Zybo Z7-20 FPGA has 40 peripheral module (Pmod) connections suitable for transmission of video data but cannot transmit all five projectors in parallel. To allow simultaneous control of all five displays through a single FPGA, the video signals are transmitted from the FPGA in five flat-panel display (FPD) Link I serialized buses that have four differential data lanes. A custom-designed printed circuit board (PCB) converts the serial, FPD Link I signal to a parallel RGB888 VGA signal, which then drives the Lightcrafter 2000 EVM.

In a commercial DLP projection system, the projector will typically undergo multiple processing steps to improve projected video image quality and/or minimize visual artifacts, which will be considered in the following subsections. In the design of a custom system for vision experiments, these processing steps may inadvertently introduce an artifact in the stimulus. To robustly design this five-primary system, each of these processing steps was identified, measured, and addressed to ensure the stimuli generated by the system were precisely known.

#### Mapping digital level to duty cycle

Each RGB primary is allocated 1 of 256 digital levels, which is mapped from the digital level to a duty cycle through the preconfigured contour mitigation table (i.e., the manufacturer’s terminology of the de-gamma curve). The contour mitigation table is an 8-bit to 12-bit look-up table, where the duty cycle presented to the DMD mirrors has 8-bit resolution. The error between the 12-bit contour mitigation table output and the 8-bit duty cycle is spread using a spatial and temporal dithering algorithm (Van Kessel et al., 1998).

**Figure 3.**
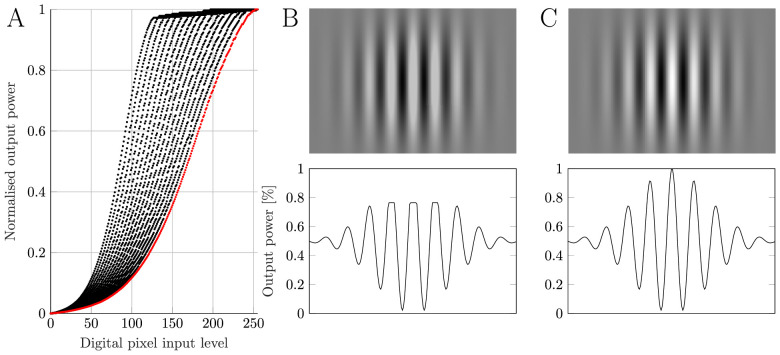
Projector gamma curve selection. (A) The measured range of gamma curves (contour mitigation tables) that map the digital pixel value to output power in the DLP. These gamma curves are selected based on the dynamic gamma curve selection algorithm in the stock DLP. The red gamma curve is always applied in this system by changing each gamma curve stored in the system’s onboard flash memory to the same curve. (B) A spatial sinusoidal stimulus pattern will undergo clipping of its peaks without compensating for the dynamic gamma curve algorithm (*upper panel:* example spatial pattern; *lower panel:* cross section of this pattern’s amplitude to highlight the clipping at pixel values). (C) By enforcing a single gamma function (red line in A), the clipping is eliminated, and a full contrast modulation is possible (*upper panel:* example spatial pattern with single gamma function enforced; *lower panel:* cross section of this pattern’s amplitude shows this clipping is eliminated).

Control of the pixel state in the stock DLP projector is augmented using an algorithm that dims the projector LEDs and proportionately increases the PWM duty cycle of the micromirrors if no more than 2,249 pixels (≈1% of pixels) within a frame are above any of the 16 threshold pixel values (where thresholds are 80 → 200 in steps of 8). It was found that this algorithm maps each input pixel value to 1 of 17 duty cycles based on which contour mitigation table is selected depending on the content of the frame (Figure 3A). Although this algorithm is undocumented in the DLP2000EVM specifications, it is thought by the authors to be designed to minimize heat in pico-projector systems.

These gamma curves were measured with an ILT1700 radiometer with 2249-pixel central stimuli that iterated in unit steps across the 256 pixel levels and a 2250-pixel corner square with a fixed pixel level to enforce a particular gamma curve. In the stock system, dimming the LED with this algorithm causes spatially small stimuli to have the upper output values clipped to below the next threshold value, and so naive control of the projector is inappropriate to generate stimuli for visual experiments. This issue is resolved in our five-primary system by reprogramming the contour mitigation table in each DLP’s flash memory so that every gamma curve is the same as the full-range gamma curve (red line in Figure 3A). As a result, clipping artifacts (Figure 3B) are eliminated, and the maximum dynamic range is available for generating a spatiotemporally modulated stimulus (Figure 3C).

#### Chromatic frame interleaving

In stock DLP projectors, all three primaries are typically modulated by one DMD, and so each primary is allocated a time slice within each 60 Hz frame (Van Kessel et al., 1998). For the DMD2000 used in this system, measurements of the current through the LEDs in the EVM show the red, green, and blue primaries are on for 40.5%, 44.5%, and 15% of a frame, respectively. To limit chromatic flicker artifacts, these time slices are interleaved through each 60 Hz video frame in a sequence of G-R-G-R-B-G-R-G-R-B. As a result of the chromatic frame interleaving, custom frame rates above 60 Hz cannot be achieved by assigning a subframe to a primary’s time slice. Therefore, to achieve higher temporal frequencies (>30 Hz), the DLP adopted in this design would have to be exchanged for a DLP with a higher frame rate option.

#### Bit-splitting

DLP projectors use PWM to control the digital pixel level. This technique relies on the frequency of the PWM being greater than the temporal resolution of the visual system, and as a result, the PWM signal is integrated to the average level across each frame (Arnold & Winsor, 1934). Fine-scale control of the micromirrors is constrained by the bit-splitting techniques designed to limit perceptual spatial and temporal artifacts during the PWM (Critchley et al., 1995; Hornbeck, 1997) by distributing the ON pulse throughout a primary’s time slice to reduce flicker between adjacent pixels, both spatially and temporally. This bit-splitting sequence implemented by Texas Instruments means that within a 60 Hz frame, the precise timing of the ON pulse of a primary cannot be determined.

### Optics

The optical system merges the five primary images onto the same optical axis in Maxwellian view. Each primary image is collimated by projecting onto an achromatic doublet lens (⌀=25.4 mm, $f_{1}=30$ mm, AC254-030-A; Thorlabs, Newton, NJ, USA) placed at one focal length from the virtual focal point of the projector. Achromatic doublet lenses are chosen to limit the effect of chromatic and spherical aberration. A 30-mm focal length ensures that the entire projected image is captured within the 25.4-mm diameter lens as determined from the throw ratio (TR=1.6) and aspect ratio ($AR=1.\bar{77}$) of the FLA2N DLP Optics Engine (Young Optics, Hsinchu, ROC) on the DLP projector, namely:

(1) $$\begin{array}{c} f_{1}=⌀\sqrt{\frac{1}{{\left(1/AR\right)}^{2}+1}}\times TR\; \end{array}$$

The collimated projection reaches a focal image plane at $\approx 3/4\;f_{1}$ from the collimating lens and then drifts out of focus past this point. To recover the focal image plane past $3/4\;f_{1}$, a second achromatic doublet lens can be placed at $2f_{1}$ and $4f_{1}$ from the original collimating lens (Figure 1) (Packer et al., 2001). This optical setup allows for nonpolarizing beamsplitter cubes (split ratio =50:50, 400–700 nm, CCM1-BS013/M; Thorlabs) to be placed between the two additional achromatic doublet lenses to merge the five primary images onto the same axis of projection. Half of the projected power is lost with each additional 50:50 beamsplitter. In the optical path (Figure 1), three of five primaries and two of five primaries will enter the eye at 25% and 12.5% of their original transmitted power, respectively. Dichroic mirrors can be used to increase the proportion of transmitted power reaching the system output if needed.

After all five primary images are merged onto a single projection axis, the image is focused to a final illuminant focal plane, where a 2-mm artificial pupil is placed at the focal point to ensure a constant retinal illumination independent of the natural pupil area. The participant views the merged image by placing their eye behind the artificial pupil (Figure 1). It is important to maintain the position of the observer in Maxwellian view to avoid image defocus and changes in retinal illumination that can occur with head movements. Temple bars, head restraint, and chinrest are used to prevent head movement in this system. Alternatively, a bite-bar could be employed. The focal length of the objective lens ($f_{obj}$) can be chosen to change the output visual angle of the system ($\theta_{H},\theta_{W}$). To find the output visual angle of the system given a focal length, the width ($W_{mm}$) and height ($H_{mm}$) in millimeters of a 360 × 640 pixel rectangular screen occupying the full ⌀=25.4 mm diameter of the circular objective lens:

$$\begin{array}{c} \begin{array}{cc} H_{mm}=\sqrt{\frac{{⌀}_{mm}^{2}}{1+{\left(W_{pix}/H_{pix}\right)}^{2}}} & W_{mm}=\sqrt{\frac{{⌀}_{mm}^{2}}{1+{\left(H_{pix}/W_{pix}\right)}^{2}}}\\ H_{mm}=12.4\text{mm} & W_{mm}=22.1\text{mm} \end{array} \end{array}$$

So that the visual angle $\theta_{H},\theta_{W}$ from a $H_{mm}\times W_{mm}$ image through a lens with a focal length of $f_{obj}=100$ mm is:

$$\begin{array}{c} \begin{array}{cc} \theta_{H}=2\tan^{-1}\left(\frac{H_{mm}}{2f_{obj}}\right) & \theta_{W}=2\tan^{-1}\left(\frac{W_{mm}}{2f_{obj}}\right)\\ \theta_{H}=7.1^{\circ } & \theta_{W}=12.6^{\circ } \end{array} \end{array}$$

This means that the projected image, which has the geometry of a pyramid with dihedral angles of $\theta_{H}=7.1^{\circ },\theta_{W}=12.6^{\circ }$, will have a output solid angle of

$$\begin{array}{ccc} \Omega \; & = & 4\sin^{-1}\left(\sin\left(\frac{\theta_{H}}{2}\right)\sin\left(\frac{\theta_{W}}{2}\right)\right)\\ \Omega \; & = & 0.0272\text{sr} \end{array}$$

This calculation can be used to determine the radiance of the image when the power of the final objective lens is changed to alter the visual angle of the stimulus field and to calculate the image size with higher- or lower-power lenses. The optical design offers the advantages that the primary LED and interference filter combinations can be easily changed as per the experimental requirements.

### FPGA design

**Figure 4.**
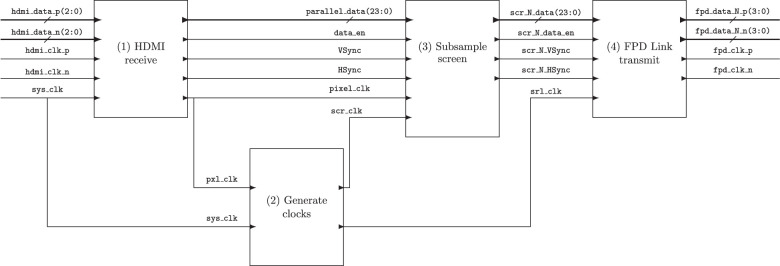
Block diagram of FPGA functionality separated into its four modules. This system receives (1) a high-definition HDMI video input and allows (2) synchronous control of five projectors by (3) subsampling a high-resolution (1920 × 1080 pixel) image frame into five lower-resolution (640 × 360 pixel) images that are synchronously (4) transmitted through five parallel FPD Link I outputs.

**Figure 5.**
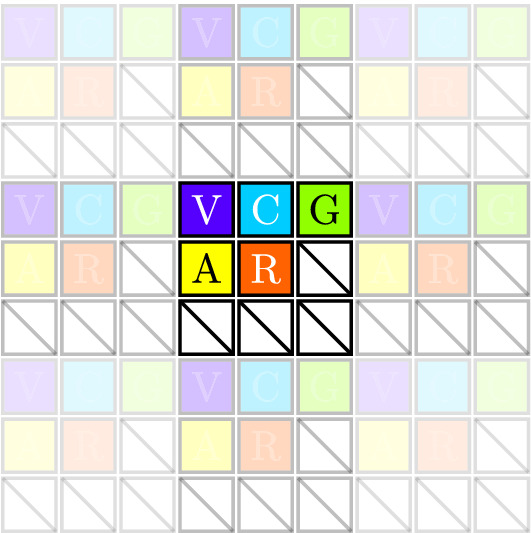
Active pixel sampling in the subsample screen module. The active data resolution (1920 × 1080) contains 9× the active pixels of the projector resolution (640 × 360). The input frame is grouped into 3×3 kernels with each of the five output frames referenced relative to that kernel. This method supports independent control of up to nine projectors using one HDMI cable.

A central Zybo Z7-20 FPGA controls the five DLP projectors. This FPGA divides a high-resolution (1920×1080 pixel), HDMI input frame into five lower-resolution (640×360 pixel) FPD Link I output frames that are transmitted to the corresponding primary projector using the four modules shown in Figure 4: (1) HDMI receive: The system receives an HDMI input signal from the controlling PC and recovers the pixel data in parallel RGB888 format. (2) Generate clocks: Takes the recovered pixel clock from the HDMI input signal and generates synchronized pixel clocks and video framing data to ensure all five projector outputs are temporally synchronous. (3) Subsample screen: Divides the high-resolution input video frame into five lower-resolution output video frames (shown in Figure 5) and reconstructs the lower-resolution VGA frame structure. (4) FPD Link transmit: Serializes the 28 parallel pixel data and control bits into four differential data lines and one differential clock line according to the FPD Link I data format specifications in order to generate five FPD Link I outputs for each projector.

The FPGA is programmed with very high-speed integrated circuit hardware description language (VHDL) in Vivado 2020.1 (Xilinx). The HDMI receive module is implemented as a block design and is built around the Digilent’s dvi2rgb intellectual property (IP) block. The digital video interface (DVI) IP recovers the phase of the transition-minimized differential signaling (TMDS) clock signal and decodes the 8b/10b encoded data signal through an in-built encoding table. The Generate clocks module is built on the MMCME_adv primitive and uses an integer clock divide of 1/9th for the lower-resolution VGA clock and a fractional divide of 7/9th for the FPD Link I output clock. Each clock is globally buffered using a BUFG primitive. The FPD Link transmit module implements a 7:1 serialization algorithm that formats the 27 parallel data and control bits into four differential data lines and one differential clock line.

Of these four modules, the subsample screen module implements the most novel functionality (Figure 5). As the output video resolution transmits 1/9th of the active pixels in each frame, the subsample screen module groups the 1920×1080 image into 3×3 kernels, resulting in 640×360 kernels. Each of the five primaries is indexed in the kernel to allow five separate lower-resolution images to be encoded in the higher-resolution frame (Figure 5). The data are also buffered in a way that allows the data to cross internal clock domains within the FPGA to retain temporal synchrony. The data are then read out of the first in, first out (FIFO) buffer while transmitting active data, with the lower-resolution blanking and synchronization pixels reconstructed around the buffered active data.

### PC software control

At the central PC, the five-primary system is connected as an external monitor. Each 1920×1080 pixel frame is a composite image of the five 640×360 pixel frames that are indexed, as shown in Figure 5. These composite video frames are constructed with a custom MATLAB script that then uses Psychtoolbox (Brainard, 1997; Pelli, 1997; Kleiner et al., 2007) to send the composite video frame to the external monitor. Alternatively, the external monitor can be presented with a video file that has been previously constructed in MATLAB to reach 60 Hz frame rates. In addition to constructing the composite video frame, the computer graphics settings for the external monitor are tuned to prevent any additional color transforms.

### Choice of primaries

Receptor silent substitution (Estevez & Spekreijse, 1982; Shapiro et al., 1996) is the technique predominantly used in psychophysical experiments to probe human melanopsin function (Tsujimura et al., 2010; Cao et al., 2015; Spitschan et al., 2017; Zele, Feigl, et al., 2018; Allen et al., 2019). Effective silent substitution of one or a combination of photoreceptor excitations requires implementation of narrowband primary lights that maximize the contrast of SMLRi excitations for a specific photoreceptor (where, S = S-cone, M = M-cone, L = L-cone, R = rhodopsin, i = intrinsic melanopsin) between a high and low condition, while maintaining the same excitations for four other unmodulated (i.e., silenced) photoreceptors between the two conditions.

The efficacy of a set of five primary LEDs for silent substitution stimuli can be quantified by the maximum achievable contrast of α-opic excitations between minimum and maximum stimuli. The α-opic power for a single primary and photoreceptor ($P_{\alpha }$) is a function of the spectral output of the LED ($P_{\lambda }$) and the α-opic spectral sensitivity function ($s_{\alpha }$), namely (International Commission on Illumination, 2018):

(2) $$\begin{array}{c} P_{\alpha }=\int P_{\lambda }\left(\lambda \right)s_{\alpha }\left(\lambda \right)\;\text{d}\lambda \; \end{array}$$

A linear combination of α-opic excitations for all five primaries will provide the total α-opic excitations ($\vec{\alpha }$) for the power output from the five primary LEDs ($\vec{p}$) (Cao et al., 2015):

(3) $$\begin{array}{ccc} & & \;\;\;\;\;\;\;\;\;\;\;\;\;\;\;\;\vec{\alpha }=A\vec{p}\\ & & \;\;\;\;\;\;\;\;\;\;\;\;\;\;\;\;\left(\begin{array}{c} \alpha_{S}\\ \alpha_{M}\\ \alpha_{L}\\ \alpha_{R}\\ \alpha_{i} \end{array}\right)=\left[\begin{array}{ccccc} a_{S,V} & a_{S,C} & a_{S,G} & a_{S,A} & a_{S,R}\\ a_{M,V} & a_{M,C} & a_{M,G} & a_{M,A} & a_{M,R}\\ a_{L,V} & a_{L,C} & a_{L,G} & a_{L,A} & a_{L,R}\\ a_{R,V} & a_{R,C} & a_{R,G} & a_{R,A} & a_{R,R}\\ a_{i,V} & a_{i,C} & a_{i,G} & a_{i,A} & a_{i,R} \end{array}\right]\times \left(\begin{array}{c} p_{V}\\ p_{C}\\ p_{G}\\ p_{A}\\ p_{R} \end{array}\right)\; \end{array}$$

where each element of the A matrix ($a_{i,j}$) represents the excitation of the ith photoreceptor as a result of a single unit of the jth primary, which was found with Equation 2. The spectral output ($P_{\lambda }$) of each primary is measured at their maximum primary output so that $\vec{p}$ is the percentage of maximum power output for each primary. The α-opic contrast between the excitations from minimum (α min ) and maximum (α max ) stimuli are found with either the Weber or Michelson contrast:

(4) $$\begin{array}{ccc} C_{\text{Weber}}\; & = & \frac{{\vec{\alpha }}_{\max}-{\vec{\alpha }}_{\min}}{{\vec{\alpha }}_{\min}}\\ C_{\text{Michelson}}\; & = & \frac{{\vec{\alpha }}_{\max}-{\vec{\alpha }}_{\min}}{{\vec{\alpha }}_{\max}+{\vec{\alpha }}_{\min}}\; \end{array}$$

where

(5) $$\begin{array}{c} {\vec{\alpha }}_{\min}=A{\vec{p}}_{\min}\;{\vec{\alpha }}_{\max}=A\left({\vec{p}}_{\min}+\Delta \vec{p}\right)\; \end{array}$$

Weber contrast is appropriate for application with aperiodic stimuli that are spatially small and/or temporally brief with reference to the adapting background field. Michelson contrast can be used with stimuli having a periodic spatial and/or temporal composition when both the maximum and minimum components contribute to the adapting field (i.e., sine wave gratings) (Peli, 1990).

Maximizing the achievable contrast for a specific photoreceptor in a silent substitution protocol requires the optimal choice of five (and no more) primaries for the test condition (Evéquoz et al., 2021). The maximum achievable contrast in melanopsin for a set of five primaries can be found with

(6) $$\begin{array}{ccc} C_{\text{Weber}}\; & = & \frac{1}{-\sum_{i=1}^{5}a_{5,i}\text{min}\left\{a^{i,5},0\right\}}\\ C_{\text{Michelson}}\; & = & \frac{1}{\sum_{i=1}^{5}a_{5,i}\left|a^{i,5}\right|}\; \end{array}$$

where $a_{5,i}$ is the row of A corresponding to melanopsin excitation and $a^{i,5}$ is the corresponding column of $A^{-1}$, which is the natural extension of the result from Evéquoz et al. (2021) from four primaries (leaving rods uncontrolled) to five primaries with full silent substitution. An intuitive understanding of this result comes from the five primary powers required to produce a silent, unit change in melanopsin excitation $\Delta \vec{\alpha }$, namely:

(7) $$\begin{array}{c} \Delta \vec{p}=A^{-1}\Delta \vec{\alpha }=A^{-1}{\left(\begin{array}{ccccc} 0 & 0 & 0 & 0 & 1 \end{array}\right)}^{T}=a^{i,5}\; \end{array}$$

As the A matrix is nonsingular, there is one scaled $\Delta \vec{p}$ to move in the silent melanopsin direction. Due to the overlapping spectral response of the five opsins, there can be no primary that can modulate the target photoreceptor orthogonal to all other photoreceptors. This means both positive and compensatory negative changes in primary powers will be needed with reference to the minimum stimuli to silently move in the melanopic direction (or other targeted photoreceptor). As negative primary powers are not possible, the minimum stimuli power ${\vec{p}}_{\min}$ must at least contain an equally positive amount of the required negative change in powers in $\Delta \vec{p}$ so that both conditions are feasible. In fact, any minimum stimuli power above this will increase the minimum melanopsin excitation without increasing $\Delta \vec{\alpha }$ and so the maximum melanopic contrast of a set of five primaries is given when

(8) $$\begin{array}{c} \;{\vec{p}}_{\min}=-1\times \text{min}\left\{\Delta \vec{p},0\right\}\;{\vec{p}}_{\max}=\vec{p_{\min}}+\Delta \vec{p}\; \end{array}$$

The melanopic contrast produced from these conditions can be rearranged to the expression given in Equation 6 and this process of finding the maximum contrast can be applied to any target photoreceptor.

Using this process to select five primaries will result in the largest contrast of the target photoreceptor but will also define the background adapting chromaticity that this contrast has to be measured at. When used for five-primary systems, this analysis alone is often insufficient because the range of background chromaticities that offer close to this peak single photoreceptor contrast is narrower than four primary systems that do not control rhodopsin excitation, and the sets of five primaries that produce the largest melanopsin contrast result in deep red background chromaticities whose melanopsin excitation is a small fraction of the total photoreceptor excitation in the background.

**Figure 6.**
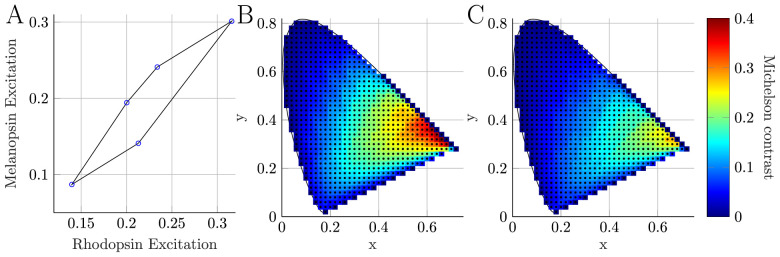
Analysis of maximum achievable contrast for single-photoreceptor directed stimuli at a specified chromaticity. (A) The convex hull representing the total gamut of rhodopsin (R) and melanopsin (i) excitations for the optimal set of five primaries (circles, summarized in Table 1) to maximize melanopsin excitation at a chromaticity of (x,y,z)=(1/3,1/3,1/3). Each vertex represents each of the (at most) five, three-primary solutions to Equation 9. Each of the line segments represent all the four-primary solutions to achieve the specified chromaticity if one primary is switched off. All five-primary solutions for the specified chromaticity are bounded by this convex hull. Because of the geometry of these convex hull polygons, the maximum silent melanopsin or rhodopsin contrast will be modulated up or down from one of the vertices and can be found according to Equation 10. (B) Analysis of the maximum melanopsin-directed, Michelson contrast (colored squares) across an equally spaced grid of chromaticities (black dots). (C) Analysis of the maximum rhodopsin-directed, Michelson contrast (colored squares) across an equally spaced grid of chromaticities (black dots).

To find the maximum single-photoreceptor contrast at a specified background chromaticity (e.g., equal energy white), we must consider the gamut of achievable rhodopsin (R) and melanopsin (i) excitations at a specified chromaticity. All Ri-excitations achievable by the five-primary system at the specified chromaticity are bounded by the convex hull formed from the Ri-excitations produced by each of the purely positive power, three-primary mixes that achieve the specified chromaticity (Figure 6), which we will refer to as virtual primaries (${\vec{p}}_{V}$):

(9) $$\begin{array}{c} \vec{p_{V}}=B_{3\times 3}^{-1}{\left(\begin{array}{c} x,y,z \end{array}\right)}^{T},\;\forall {\vec{p}}_{V}\in R_{\geq 0}\; \end{array}$$

In this analysis, the background chromaticity will be set using the CIE $10^{\circ }$ physiologically relevant XYZ functions (International Commission on Illumination, 2015). To find these virtual primaries, a matrix $B_{3\times 5}$ can be constructed that relates the power of each of the five primaries’ contribution to XYZ, and $B_{3\times 3}$ is the corresponding B matrix for each of the permutations of three primaries that can achieve the specified chromaticity. If X+Y+Z=1, then XYZ = xyz with no loss in the generality as the solution can be rescaled without changing achievable contrast.

The maximum single-photoreceptor contrast at a specified chromaticity can be found from the convex hull of the Ri-excitations (Figure 6). For a maximal contrast stimulus at this chromaticity, either the minimum or maximum stimulus will be one of the virtual primary vertices depending on whether the vertex is on the upper or lower bound of the convex hull. The other stimuli condition (${\vec{p}}_{\mathrm{mod}}$) can be found by scaling $\Delta \vec{p}$, namely:

(10) $$\begin{array}{ccc} {\vec{p}}_{\mathrm{mod}} & = & {\vec{p}}_{V}+c_{1}\Delta \vec{p}\\ c_{1} & = & \text{min}\left\{-\left.\frac{p_{V}}{\Delta p_{i}}\right|\Delta p_{i}<0\right\}\\ & - & \text{min}\left\{\left.\frac{p_{V}}{\Delta p_{i}}\right|\Delta p_{i}>0\right\}\; \end{array}$$

where $c_{1}$ scales the $\Delta \vec{p}$ to ensure the primary powers are nonnegative at both conditions. The contrast for each of these modulations from a virtual primary can be found in Equation 4 and the maximum contrast achieved by one of these modulations will be the maximum photoreceptor-directed contrast at that chromaticity for the five-primary system.

In choosing a set of custom illuminants for the five primaries of the proposed display, we aimed to optimize the achievable contrast across all photoreceptors. As melanopic and rhodopic vision have higher contrast thresholds than cone-mediated vision (Zele & Cao, 2015; Lamb, 2016; Zele, Feigl, et al., 2018; Uprety et al., 2022), this optimization prioritized maximizing the achievable melanopsin and rhodopsin contrast. Consideration was also taken to ensure the maximum melanopsin contrast stimuli of the chosen five primaries did not have a deep red background chromaticity. To start this analysis, both a set of potential LEDs and narrowband filters will need to be selected for the display.

The LUXEON Rebel Color LED line (Lumileds, San Jose, CA, USA) was chosen for its range of high-power LEDs with peak wavelengths spanning the visible spectrum. Thorlabs visible spectrum bandpass filters (Thorlabs) with a full width at half maximum (FWHM) bandwidth of 10 nm were chosen to narrow the bandwidth of the LEDs and to push each primary’s photoreceptor excitations closer to the spectrum locus and increase the available gamut of the chosen set of five primaries. In this analysis, all bandpass filters with a center wavelength between 400 and 650 nm were analyzed (being the range of spectral sensitivity of all five photoreceptors). These center wavelengths were separated by 10 nm across this range.

**Figure 7.**
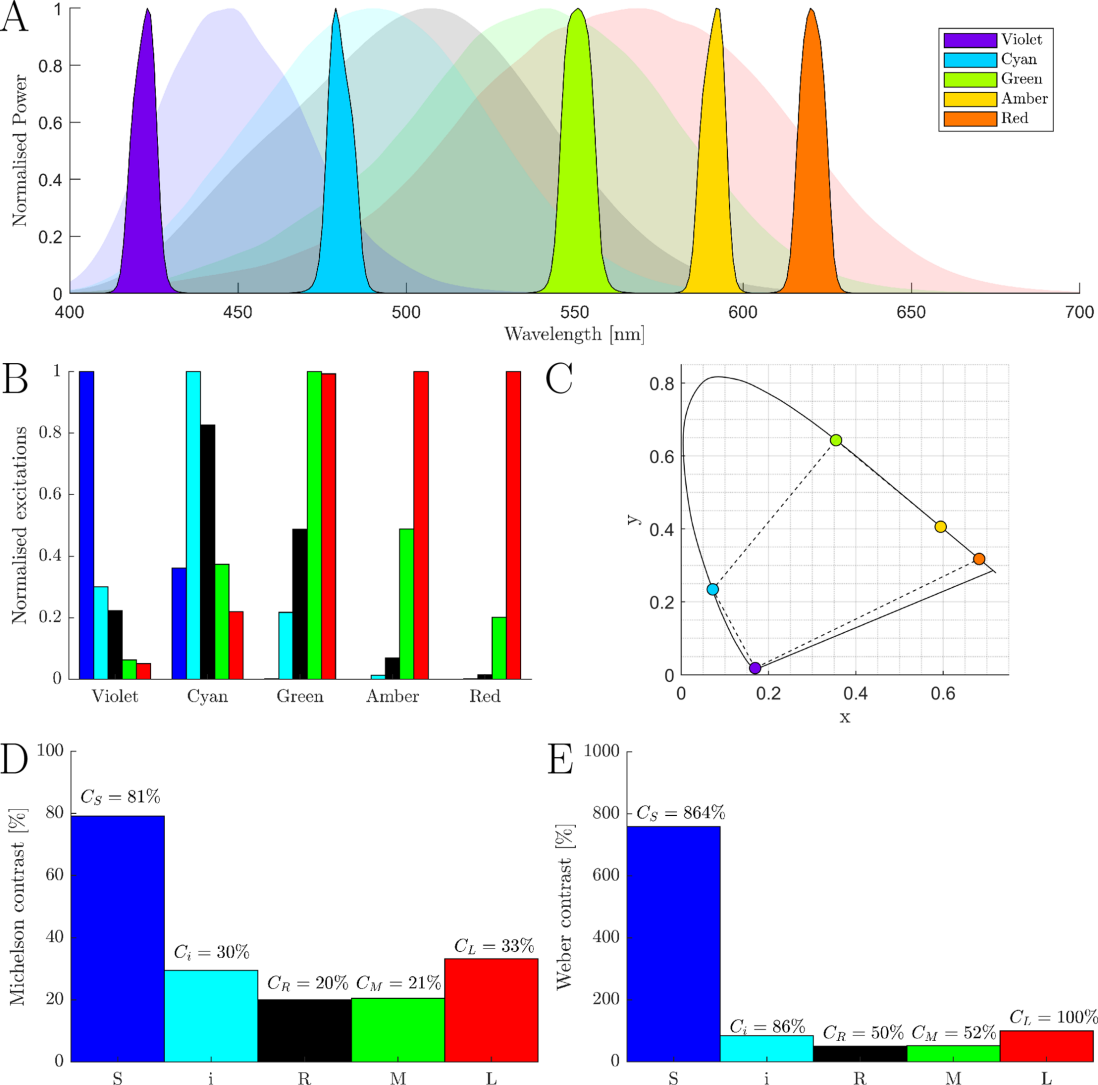
Analysis for the selection of the five primary LED and narrowband filter combinations. (A) The five primaries (opaque curves) chosen to optimize the maximum achievable melanopic and rhodopic contrast. For each primary, the LUXEON Rebel Color LED and Thorlabs bandpass filter (FB) combination is Violet: Violet + FB420 nm, Cyan: Blue + FB480 nm, Green: Lime + FB550 nm, Amber: PCAmber + 590 nm, and Red: Red Orange + FB620 nm. Maximum irradiance output measured from the system’s pupil (Violet: 0.82 [W/m${}^{2}$], Cyan: 0.54 [W/m${}^{2}$], Green: 0.35 [W/m${}^{2}$], Amber: 0.42 [W/m${}^{2}$], Red: 0.16 [W/m${}^{2}$]). The α-opic spectral sensitivity functions (transparent curves) are shown with the primary lights. (B) The α-opic excitations of the five primaries. These are the column values from the A-matrix in Equation 3 (S-cone = blue; melanopsin = cyan; rhodopsin = black; M-cone = green; L-cone = red). (C) Chromaticity coordinates of the five primaries specified using the CIE $10^{\circ }$ physiologically relevant XYZ functions. (D) Maximum achievable Michelson contrast during photoreceptor-directed silent substitution (Equation 4). (E) Maximum achievable Weber contrast during photoreceptor-directed silent substitution (Equation 4).

With the spectral power output for all LUXEON Rebel Color LEDs and the spectral transmission for Thorlabs 10-nm FWHM bandpass filters, a MATLAB (version 2019b; MathWorks, Natick, MA, USA) script was written to compare the achievable contrast for each photoreceptor for the (285)=98,280 permutations of highest output LED and filter combinations. Without constraint to the maximum LED power or background chromaticity, a melanopic Michelson contrast of 44% (with a rhodopic Michelson contrast of 27%) can be achieved. However, these contrasts rely on narrowband primaries at the extremes of the S- and L-cone sensitivity functions, which required large relative primary powers and with a deep red adapting chromaticity. To constrain this analysis, we only considered sets of primaries that produced a maximal stimulus across a range of chromaticities that were not a deep red. The set of primaries chosen (Figure 7) was capable of producing a 30% melanopsin and 20% rhodopsin contrast at an orange adapting chromaticity of (x,y,z) = (0.5,0.45,0.05). In addition to the set of five primaries presented here, we also followed the algorithm outlined above to determine the set of five primaries that maximize the melanopsin- and rhodopsin-directed contrast with an equal energy white chromaticity (Table 1).

**Table 1.** Primaries from the LED and filter combinations considered, which provide the maximum contrast for melanopsin and rhodopsin at a target chromaticity of (x,y,z)=(1/3,1/3,1/3).

Target opsin	Peak wavelength [nm]					Max. Michelson contrast	
Melanopsin	400	470	538	570	654	R: 11.17%	i: 20.28%
Rhodopsin	400	470	530	570	654	R: 13.45%	i: 18.92%

### General methods

The system was calibrated and its performance evaluated through the following procedures: (1) alignment of each DLP in six dimensions; (2) spatial homogeneity measurement and correction; (3) measurement of the spectral and radiometric outputs of each primary, including their warm-up and output stability characteristics; (4) validation of the temporal synchronization of the projectors; and (5) photographing a structured five-primary rendered color image through the artificial pupil.

Radiometric measurements were completed with an ILT1700 radiometer and photometer (International Light Technologies Inc. Peabody, MA). Spectral measurements of the system were performed with the EPP2000C-50 µm Slit UV-VIS Spectrometer (StellarNet, Tampa, FL, USA). Measurements of the temporal response of the system were measured with a PIN silicon photodiode and digitally acquired at 200 k samples/s with a Power Lab 4/30 (ADInstruments Pty Ltd, Sydney, Australia). Stimuli were generated and measurements analyzed in MATLAB 2019b (MathWorks).

## Results

### Projector alignment

**Figure 8.**
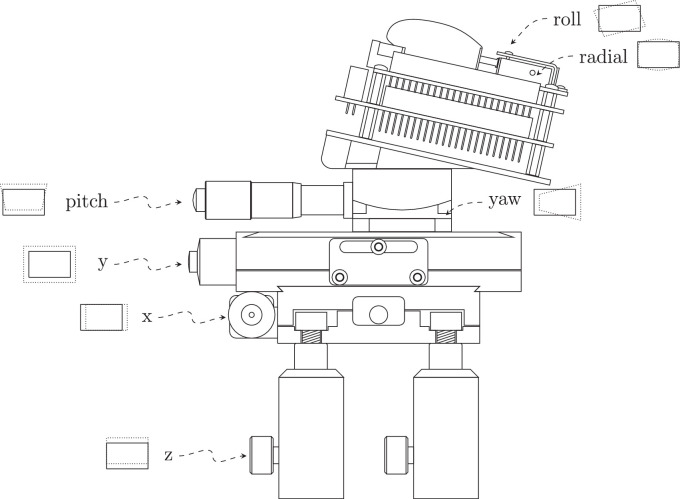
Side profile of a single primary projector mounting and alignment stage and the tools used to align the six degrees of freedom for each primary. Each alignment tool is labeled with which type of alignment it controls, including an indicative representation of how it changes the final projected image for that primary. The mounting and alignment stage is pictured in Figure 2B.

Merging five projections onto the same optical axis to achieve pixel-level control of the photoreceptor excitations requires precise alignment of all five projectors. For this purpose, the position of each projector can be altered across six degrees of freedom (Figure 8), where the mounting used for each projector has fine-scale control of three degrees (x, y, and pitch) and course control of the remaining three degrees (z, roll, and yaw). Each projector is mounted on two linear translation stages (XR25P/M; Thorlabs) to achieve micrometer control of the x and y position. The vertical position (z) of the projector is adjusted by the position of the set screw in the four mounting posts. The pitch of the projector is controlled by a goniometer (GNL10/M; Thorlabs) in addition to a custom three-dimensional printed mounting wedge with a constant $12.5^{\circ }$ pitch angle offset to account for the pitch of the DLP’s projection. Although the remaining two degrees of freedom (roll and yaw) are mostly fixed in this system, a minor degree of roll alignment ($<5^{\circ }$) is handled by adjusting the mounting screw tightness on either side of the optical engine, and the yaw alignment ($<5^{\circ }$) is controlled based on the angle in which the goniometer is mounted to the translation stage. The series of achromatic doublets necessary to merge the five projectors (Figure 1) introduce some radial distortion into the system. The nature of this radial distortion can be partially controlled (but not eliminated) by changing the y-position of the projector relative to its first achromatic doublet and by changing the focus pin on the projector’s optical engine.

A systematic calibration process was taken to achieve optical alignment of each projector in the eye of an observer in Maxwellian view. During this process, a grid pattern of six, equidistant, single-pixel lines spanning the image in the horizontal and vertical directions was presented to the observer. A reference primary was chosen for alignment of the other four projectors. This reference primary was centered within the lenses in the optical path to remove de-centering distortions and projected square onto the retina to remove keystone distortion. Depending on the y-position and focus pin position of the projector, the radial distortion of this reference primary could be changed from a monotonic, negative radial distortion (barrel distortion) to a complex radial distortion (moustache distortion). A barrel distortion was chosen as it simplifies the quantification of the radial distortion to allow a preprocessing algorithm to remove it from the projected image.

Once the reference primary’s position is optimized, the other four primaries are each aligned against this reference primary. The first step is to ensure the primary has the same radial distortion as the reference. Changing the focal pin position of the projector’s optics provides coarse control of both image magnification and radial distortion of the image. Adjusting the y-position of the projector changes the image magnification with minor changes in the radial distortion. The focal pin of each projector is set to match the radial distortion of the reference, and then the y-position is set to match its magnification. The second alignment stage corrects the roll alignment by turning the mounting screws on the optical engine. This lowers or raises the height of one side of the projected image relative to its other side by tightening/loosening the mounting screws on the respective sides. The third stage aligns both the x-position and yaw of the projector, whereby the yaw angle would be coarsely aligned and then a fine-scale alignment of x-position would achieve pixel-level alignment in the x-axis of the image. The final alignment stage sets the z-position and pitch alignment of the projector. Because the set screws of the mounting posts only provided coarse control of the z-position of the projectors, the z-position was coarsely aligned and then the goniometer achieved pixel-level alignment by varying the pitch. Due to having only coarse control of some degrees of freedom, this alignment procedure was an iterative approach, where a small misalignment of a certain degree of freedom only became apparent after improving the alignment in other dimensions.

We typically completed this calibration procedure with two people working in combination, with one observer instructing the second person on the direction of changes needed in the alignment components. Pixel-level, optical alignment of the five projectors was confirmed by eight observers who were members of the laboratory. These observers were presented both the grid image used for alignment, along with a complex, grayscale video, and were instructed to look for any chromatic aberration in the image. All observers reported that there was no chromatic aberration or motion artifacts in the image. Once the system has been aligned, it will only need realignment if there is a component change in the system (e.g., a different LED and interference filter combination or different final objective lens).

### Spatial homogenization

**Figure 9.**
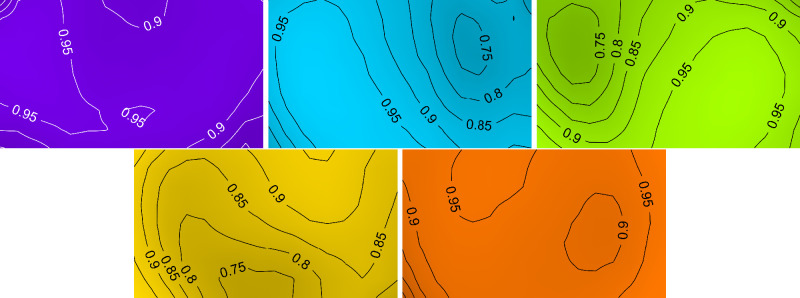
Measured inhomogeneity of the five primaries during the spatial homogenization procedure. These spatial inhomogeneity measurements are inverted to create a digital mask that was psychophysically scaled to achieve a homogeneous projected image. Image order left to right, top to bottom (V, C, G, A, R).

The spatial distribution of the spectral power of the five primaries needs to be calibrated to ensure the power of each primary is known at each pixel. The custom optics built into the Young Optics engine (inset, Figure 1) are designed to focus the LED power on the DMD while spatially diffusing the power output from the LED and uniformly illuminating the DMD. Nevertheless, the directional nature of LEDs means that the spatial power distribution of each primary differed across the projected screen. To quantify this inhomogeneity, we divided the screen into 16 × 9 equal-sized squares (40 pixels × 40 pixels) and the local output radiance of each square was measured in the plane of the artificial pupil with the radiometer. After accounting for the directional sensitivity of the sensor, a spatial map of output intensities for each pixel was created by interpolating between each measurement position (Figure 9).

To account for the spatial variation of LED power across each projector’s screen, we applied a spatial weighting mask constructed by inverting the spatial map of output pixel intensities. We then subjectively reviewed the measured masks by schematically drawing the appearance of the inhomogeneity map for each primary and confirmed that they followed the same spatial pattern as in the radiometrically measured maps (Figure 9). The raw weighting masks overcorrect the spatial inhomogeneity in the projected images so that the screen inhomogeneity inverts when viewed by an observer. We therefore psychophysically rescaled the masks by linearly expanding or contracting the pixel weights until there no perceptible spatial inhomogeneities in each projector.

### Primary output validation: Spectrum, irradiance and chromatic reproduction

The normalized spectral output of each primary is shown in Figure 7A and the measured maximum radiance output per primary is presented in the caption. As the projectors use DLP technology, there is no concern with phosphor constancy at different levels because digital light levels are controlled independently to the LED by the digital micromirror array. The five primaries were linearized through a look-up table (LUT), which maps a floating point pixel value to a set of RGB levels, which generate the nearest output power. This linear gamma curve is confirmed by measuring the radiometric output of each primary for each of the 756 output levels. The warm-up and stability of the five-primary outputs were measured with the radiometer from start-up during 40 minutes of continuous operation. The primary outputs remained constant after 10 minutes.

**Figure 10.**
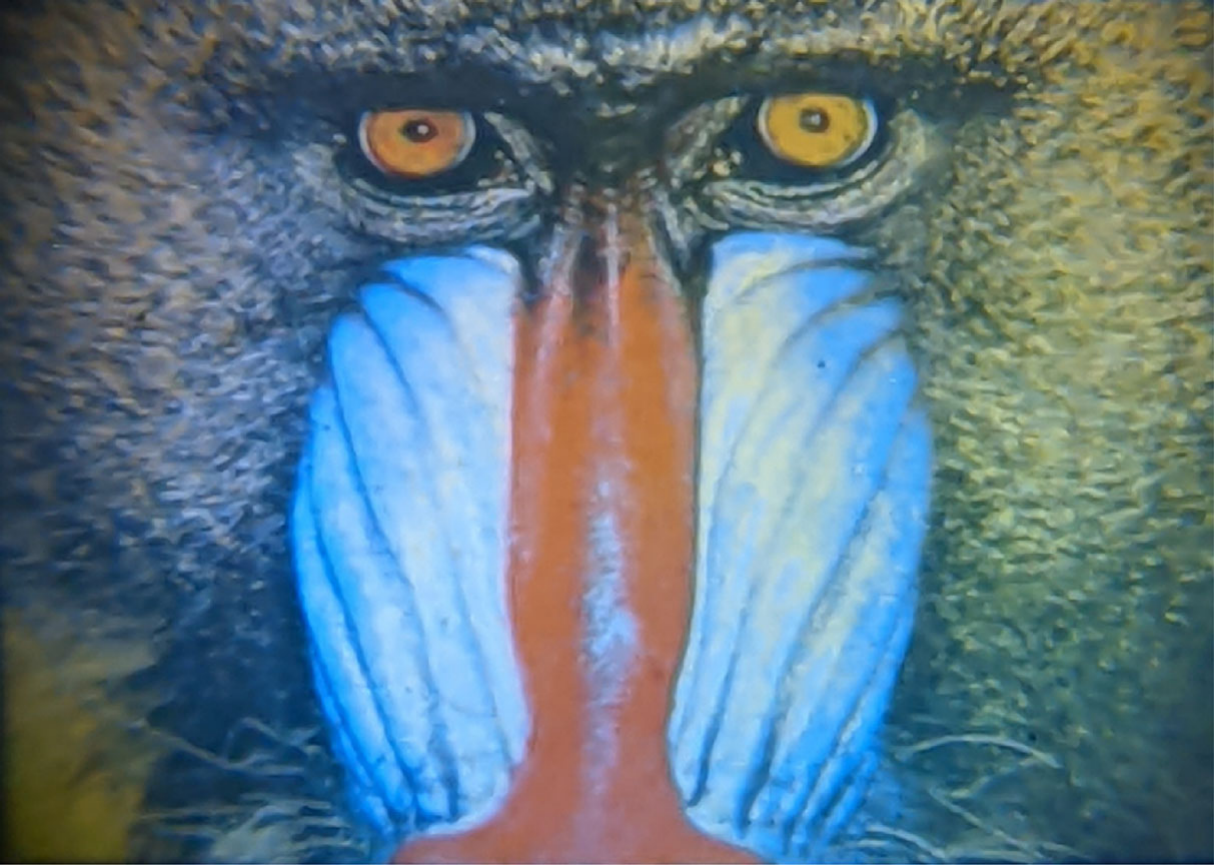
Image of an Old World monkey (*Mandrillus sphinx*) projected through the five-primary system and photographed at the 2-mm artificial pupil. Each pixel chromaticity is constructed with a combination of all five primary outputs. This image is only indicative of the actual output because the smartphone camera cannot accurately render the true color appearance of the image.

The confirmation of the spatial homogeneity, spectral content, and irradiance of the five-primary images allows a color image to be projected through the system with the photoreceptor excitations of each pixel completely specified. To demonstrate this, a test RGB image of a mandrill was converted to cone excitations using three artificial, Gaussian primaries with the sRGB chromaticity coordinates. The five primary powers needed to achieve the calculated cone excitations of each pixel were found using the $A^{-1}$ matrix for the five primaries in the system. This image was projected through the system using the procedures described above and photographed (Figure 10) through the artificial pupil. For an experimental stimulus that contains complex spatial patterns such as a natural image (Figure 10), the achievable photoreceptor-directed contrast will be chromaticity dependent (Figure 6). This means that a modulation of a photoreceptor excitation between two states will vary in contrast across the image. Equations 9 and 10 can be used to define the range of photoreceptor excitations achievable at each pixel chromaticity, and the descriptive statistics of excitation changes across the image can be reported.

### Temporal validation

**Figure 11.**
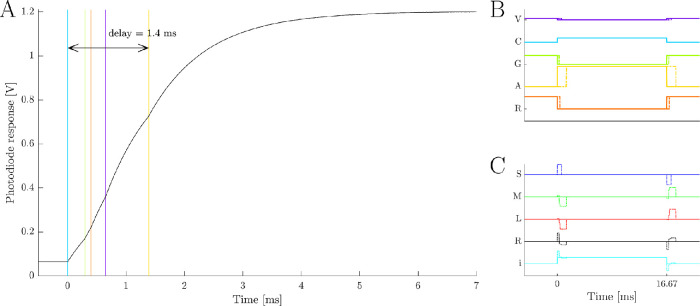
Measured temporal synchrony between the five primary image planes. (A) The temporal delay is defined as the time from the photodiode response to the transition from an OFF to ON image frame. As the response time of the photodiode is close to the delay between primary image planes, the transition to an ON image frame is measured from the inflection point of the exponential response of the measured photodiode voltage. The delays were subsequently confirmed by measuring the timing between the violet primary and each of the remaining four primaries individually. (B) Example primary powers required to generate a maximum contrast step change in a melanopsin-directed stimulus over one 60 Hz frame (16.67 ms) while keeping all other photoreceptor classes silent. The solid line is the ideal response with no temporal misalignment; the dashed line is the actual response with the measured misalignment. (C) The resulting photoreceptor excitation Weber contrast for the example silent substitution protocol in (B). The temporal misalignment of the primaries introduces a ≤ 1.4-ms rectangular pulse artifact in the intended silent substitution stimuli.

A multiple projector solution for five-primary silent substitution stimuli needs all projectors’ frames to be temporally aligned so that visual artifacts are not present at stimulus onset and offset between frame transitions (Figure 11). A single-photoreceptor directed stimulus requires a simultaneous change in primary powers to silently generate a step change in excitation (Figures 11B, C, solid lines). Temporal misalignment (Figures 11B, C, dashed lines) will introduce a rectangular pulse artifact at the frame transitions that may (or may not) be detectable to the silenced photoreceptor classes. To ensure these artifacts are not perceptible, the temporal misalignment must be narrow enough to ensure the energy in the rectangular pulse is imperceptible under the viewing conditions (Bloch, 1885).

To quantify the temporal alignment, the timing of the five primaries at onset of an all OFF- to ON-frame transition (zero to maximum output) was recorded with a precision digital scope meter (200 k samples/s) connected to a photodiode positioned at the artificial pupil (Figure 11A). The measured frame timing was confirmed by measuring each primary’s timing relative to a chosen reference primary, which also demonstrated the temporal alignment of each primary was static across frames. An ideal photodiode response would produce a step change in recorded light output at the onset of each projector’s frame. Because the photodiode used has some capacitance, the transient photodiode response to a step change in light is a decaying exponential with a time constant of τ = 955 µs. With this type of transient response, the recorded frame onset times are defined by the five inflection points of the photodiode’s decaying exponential response to all projectors switching on at the same frame (Figure 11A). We observed that the temporal alignment between projectors is consistent over multiple frames and has a pulse width of roughly 1.4 ms, which will introduce photoreceptor contrast artifacts (Figure 11C) well below the critical duration (Bloch, 1885). It should be noted that the photoreceptor contrast of the erroneous artifact generated by temporal misalignment can be minimized by the choice of stimuli. For example, stimulus waveforms presented as temporal step changes (Figure 11C) will generate the maximum erroneous contrast, while waveforms with a windowed onset and offset (e.g., cosine envelopes) are less susceptible to temporal artifacts because the error is distributed over many frames. With reference to classic temporal and summation data (Barlow, 1958), for a foveally presented photopic stimulus pulse with a size larger than the critical summation area, the 1.4-ms temporal artifact would need an intensity >1.5 log units above that intensity required for threshold detection measured with stimuli longer than the critical duration. The measured temporal misalignment is therefore unlikely to influence detection threshold.

With the interprimary frame alignment of the system confirmed, a temporally modulated stimulus could be sent through the system. A Gaussian-enveloped, stationary sinusoidal grating was procedurally generated in real time and projected using the Psychophysics toolbox in MATLAB. Various spatial and temporal frequencies for the grating were tested with an observer viewing the video. The video appeared smooth to the observers and no frame drops were detected during the test.

## Discussion

We describe the development of a Maxwellian view display having independent, spatiotemporal control of five narrowband primaries. The primaries can be customized to a user’s requirements, both in peak wavelength and spectral composition, to allow the system to be optimized for different species or different photoreceptor targeted experiments. A systematic approach is detailed to select the optimum five primaries from a set of commercially available LEDs and/or narrowband interference filters, which will maximize the available contrast in photoreceptor-directed experiments. With the designed system, each primary can represent 756 unique levels for each pixel over a 60 Hz, 360 × 640 pixel video frame. To simultaneously control the five projectors in the system, the FPGA controller was designed to allow the system to be natively controlled through any computer that supports a single HDMI output. The spatial control of this system allows 230,400 individual silent substitutions across a retinal area that is size customizable by changing the final objective lens of the optical system.

With the continued development of four or more primary displays to allow receptor silent substitution, it is necessary to develop novel control systems that can independently operate each of the primary image planes. Video standards only control three pixel values per output. To extend these current standards to control more than three primaries, there are three options, namely: (1) Run multiple projectors as multiple display outputs, (2) have a fully customized output standard, or (3) embed the primary data for more than three primaries in a single video output. Here we use the latter method to embed the primary data for all five outputs within a single high-resolution video frame. The PC output arranges the high-resolution video stimuli into the 3×3 kernels (Figure 5). This output is decoded on a custom PCB and FPGA board into five, independent, lower-resolution video stimuli to drive the five projectors. Embedding multiple primaries into a single output allows more projectors to be controlled that display outputs—with simultaneous control of up to nine 360×640 pixel projectors in this system with the single HDMI 1080×1920 pixel output. This means that each primary can be controlled by a dedicated projector, and the number of levels per pixel per 60 Hz frame can be expanded from 256 levels to 768 levels (or ≈ 9.5 bits of control). Expanding the number of levels for each primary allows the system to probe just noticeable differences (JNDs) between levels over a larger dynamic range than systems with 256 levels of pixel control (Table 2, levels/pixel/primary/60 Hz frame).

**Table 2.** Summary of four or more primary systems used to generate photoreceptor-directed spatiotemporal visual stimuli. The six systems listed by the lead author are as follows: Nugent (system in this article), Yamaguchi (Yamaguchi et al., 2008), Bayer (Bayer et al., 2015), Allen (Allen et al., 2019), Hexley (Hexley et al., 2020), and Lee (Lee & Ripamonti, 2022).

	Nugent	Yamaguchi	Bayer	Allen	Hexley	Lee
Number of primaries	5	6	4	4	6	5
Peak wavelengths [ nm ]	420, 480, 550	438, 483, 524	455, 507	444, 485	444, 484, 486	437, 462, 539
	580, 620	547, 610, 659	559, 624	553, 605	496, 552, 615	588, 625
FWHM [ nm ]	9, 10, 10	15, 37, 26	24, 34	21, 26	47, 20, 20	16, 29, 25
	9, 9	42, 37, 61	48, 18	22, 69	19, 60, 99	17, 21
Peak primary radiance $\left[{W/m}^{2}/\mathrm{sr}\right]$	32, 21, 14	unspecified	unspecified	0.58, 0.36	2.5, 0.013, 0.65	3.7, 3.8, 0.34
	16, 6			0.62, 0.60	0.76, 4.1, 3.3	0.13, 1.1
Max modulation frequency [ Hz ]	46,080	15,360	46,080	92,160^a^	46,080/184,320^a^^,^^b^	24,000
Levels/pixel/primary/60 Hz frame	756	256	256	256	256/1024^a^^,^^b^	≤ 400
Frame rate [ Hz ]	60	60	60	120^a^	30/60	N/A
Resolution [ pixels ]	360 × 640	1024× 1280	912 × 1140	768 × 1024	768 × 1024^b^	≤ 912 × 1140^c^
View	Maxwellian	Newtonian	Newtonian	Newtonian	Newtonian	Newtonian
Max 4 photoreceptor contrast^d^ [%]	100, 56, 64	99, 71, 82	82, 36, 48	68, 43, 52	70, 36, 48	46, 49, 67
Order: S, M, L, R, i	65, 76	74, 89	52, 36	54, 39	35, 47	37, 28
Max 5 photoreceptor contrast^d^ [%]	81, 21, 33	93, 38, 69	N/A	N/A	52, 3, 15	47, 26, 41
Order: S, M, L, R, i	20, 30	20, 28			1, 1	15, 16

a Values are unspecified in their studies and so the largest value from the specified hardware’s data sheet is included here; this does not mean these systems are operated at these values.

b The Hexley system projects two DLPs with a resolution 768 × 1024 onto a LCD screen with resolution 2048 × 1536, where the DLP projection is diffused so that the spatial power of a single pixel has a standard deviation of 8 pixels; reported values are in the order of LCD/DLP.

c The Lee system (Cambridge Research Systems Ltd. Rochester, England) has a DLP resolution of 912 × 1140 with each mirror changing states at 6000 Hz augmented with a spatially homogeneous modulation of the primary LEDs four times per mirror state. Due to the design of their five-primary system, images have to be constructed using layers of binary image structures. Because each image structure occupies a mirror state, the spatial complexity of projected images comes at the cost of temporal and pixel value resolution. Moderately complex spatial images (e.g., sinusoidal gratings) would take longer than the integration time of the eye to construct with binary image structures.

d Michelson contrast is used for all comparisons. Maximum 4 photoreceptor contrast [%] values leave the rhodopsin (R) excitation unconstrained for S, M, L, and i values and the melanopsin (i) excitation unconstrained for R values.

The advantage of our approach compared with existing multiple display solutions (Yamaguchi et al., 2008; Bayer et al., 2015; Allen et al., 2019; Hexley et al., 2020; Lee & Ripamonti, 2022) is that the FPGA is a deterministic system that performs frame timing alignment onboard the FPGA prior to driving each projector, whereas the multiple display outputs of a graphics processing unit (option 1, above) will not have their respective frames aligned with frame timings varying on a frame-to-frame basis. This makes multiple display controllers particularly susceptible to the spurious, temporal step artifacts that may not be silent to the unmodulated photoreceptors (Figure 11). Systems with significant interprimary, temporal misalignment can mitigate temporal step artifacts by windowing the stimuli onset and avoiding high temporal frequencies that approach half the frame rate of the system.

The projectors used in this display have a 60 Hz frame rate, which can be used to represent up to a 30 Hz temporal modulation. This temporal control is sufficiently high to investigate human melanopsin function, which has a lower temporal resolution than the rod or cone pathways (Zele, Adhikari, et al., 2018; Uprety et al., 2022). Although this system implements an inexpensive projector evaluation module, the design principles extend to projectors with higher frame rates, resolution, and color bit depth. There exist potential future development opportunities for this system. The spatial and temporal resolution of the system could be increased by using the methods described here to merge projectors with higher performance specifications (i.e., pixel resolution, frame rate, or pixel bit depth). With increased performance specifications, the communication standard required to embed multiple display outputs will need at least twice as many pixels as the display. For example, two three-primary 4K displays will require a graphics processing unit (GPU) display output with 8K resolution at the same frame rate. These high data rate display outputs also require precise PCB design and higher FPGA clock rates or wider input/output busses.

The system’s optics were developed to present each projector along a common optical pathway in Maxwellian view (Westheimer, 1966). This optical design significantly increases retinal illuminance when compared with Newtonian view displays (Table 2, Peak primary radiance), which is an advantage for experiments evaluating melanopsin and rhodopsin mediated function, their interactions, and adaptation properties in photopic lighting. The more efficient conversion between LED output power and retinal illuminance results in greater thermal stability of the LEDs and projectors. Therefore, three of the same primaries can be incorporated into a single projector to triple the output power if needed while still retaining the same bit control. By optically imaging the stimulus in a fixed 2-mm artificial pupil, the melanopsin control of image- and non-image-forming functions can be studied independently of the effects of the melanopsin activation that drives the pupil to a constricted state (Gamlin et al., 2007; McDougal & Gamlin, 2010; Adhikari et al., 2015; Barrionuevo & Cao, 2016; Zele et al., 2019a) and inadvertently decreases the retinal illuminance and modifies retinal adaptation. In Newtonian view systems, pharmacological pupil dilation or a fixed artificial pupil is required to eliminate this potential confounding factor. The modular design of the system’s optics also allows the custom control of the final objective lens focal length to change the visual angle of the retinal image, while also rescaling the pixel size in terms of its visual angle. The project alignment procedures can also be used to calibrate the system in Newtonian view.

The spatial homogeneity of the DLPs and optical system have a similar spatial power distribution across the image plane (Figure 9) as reported previously (Bayer et al., 2015). Prior to application of the homogenization procedure, the spatial power distribution of the pixels varied across the display by a maximum of 29% across the screen. Any uncorrected spatial inhomogeneity will introduce inadvertent errors in the pixel-level silent substitution because the physical lights would differ from the theoretically required lights at each location. By psychophysically scaling the radiometric spatial map, we ensured the projected image was homogeneous when viewed by an observer in Maxwellian view. This correction rescaled each pixel output to match the lowest radiometric output pixel, which reduces the bit control to 545 levels per pixel per primary (9.1 bit) on the worst-affected pixel within a frame.

The width and separation of the primaries in a system directly influence the photoreceptor contrast and gamut. We evaluated the optimal combinations of commercially available narrowband interference filters and high-power LED primaries to determine the optimal primary set to maximize the melanopsin excitation while controlling the other four photoreceptors in silent substitution (Figure 7). We also show how to optimize the primary choice should a user require a specific adapting chromaticity (e.g., equal energy white). The set of five primaries presented here has been chosen to maximize the melanopic and rhodopic contrast in silent substitution stimuli at an orange adapting background (x,y,z) = (0.5,0.45,0.05). This primary choice allows higher melanopic (30%) and rhodopic (20%) contrast than existing four or more primary, spatial systems (Table 2, Max 5 photoreceptor contrast)—with the six-primary, Yamaguchi system performing comparably. A melanopsin contribution to human vision is first evident in mesopic illumination, between 20 and 200 photopic Td, such that all five photoreceptor classes are operational (Zele, Feigl, et al., 2018; Zele et al., 2019b). Four-primary systems only produce a silent, melanopsin-directed stimulus if the rod photoreceptors are completely saturated in the photopic measurements conditions. There is a growing body of evidence that suggests that rod photoreceptors continue to signal at photopic light levels in humans (Kremers et al., 2009; Shapiro, 2002; Sharpe et al., 1989) and mice (Tikidji-Hamburyan et al., 2017) in photopic illuminations common to many silent substitution experiments. In a rod-directed, silent substitution protocol with five-primary lights, Uprety et al. (2022) measured the rod response at all photopic light levels up to the instrument's limit (8,000 photopic Td) and showed that supplemental rod contrast in a melanopsin-directed stimulus (which will occur in a four-primary system) changed the temporal response characteristics from the low-pass, melanopsin response toward the bandpass, rhodopsin response. Consequently, four-primary systems do not have enough primaries to independently control the excitations of the five photoreceptor classes and so risk confounding photopic rhodopsin with melanopsin function. It should be noted that four-primary stimuli that produce a maximum contrast for rhodopsin or melanopsin will also produce a maximum contrast for the other uncontrolled photoreceptor.

The method of constructing independent primaries is important when designing a four or more primary system. For an effective primary choice, it is recommended that both custom illuminant and filter combinations are chosen to construct each primary as was done here. On the other hand, if stock RGB illuminants (e.g., LED triplets) or stock RGB filters (e.g., LCD) are used to construct the four or more primary system, the choice of primary spectra will be limited by the shape of the existing RGB spectra, which are not designed for silent substitution protocols. This then constrains the maximum, single photoreceptor-directed contrast and the background adapting chromaticity of that maximum contrast stimulus. Systems that filter multiple illuminants through three existing filters (like an LCD) have the additional risk of creating primaries that are effectively, linearly dependent because the three primary filters will dominate the shape of the primaries and will result in performance equivalent to a lower primary system.

Due to the presence of individual differences between observers in their prereceptoral filtering and photoreceptor spectral sensitivities, the method of silent substitution cannot rely on the CIE S026/E:2018 standard observer functions to produce truly silent photoreceptor-directed stimuli. An individual observer calibration should be performed to minimize these differences (Uprety et al., 2021). For a system with spatial control of the stimulus, one method is the minimum motion technique (Anstis & Cavanagh, 1983), which would be repeated for each of the five primaries.

In conclusion, our method and system introduce a novel deterministic control protocol that allows synchronous control of up to nine displays with the necessary bit depth to probe threshold-level vision. Unique to this design is the opportunity for a user to easily integrate different primary wavelength combinations and to optically modify the retinal stimulus size, with the design principles and calibration procedures suitable for other (larger) projector types having higher frame rates and pixel densities. The computational algorithm optimizes the primary wavelength combinations required for a particular experimental purpose, including with species having different photoreceptor classes, or humans with different photoreceptor spectral responses (e.g., anomalous trichromats, tetrachromats). Additional primary channels can be incorporated into the optical set-up when studying animals with more than five photoreceptor classes (e.g., avian). The five-primary display provides fine spatial control (Figure 10) of photoreceptor-directed light for application in psychophysical methodologies, pattern and multifocal electroretinograms, visual evoked potentials, electroencephalograms, multifocal pupillometry, and functional magnetic resonance imaging, and can be integrated into microscopes for physiological experiments. Together, this system allows the study of the spatio-temporal and chromatic response properties of visual and nonvisual processes (e.g., effects of light on circadian and sleep function, pupilliary control pathways, mood, alertness) when driven by a single photoreceptor class such as via the melanopsin-expressing ipRGC pathway or the interactions between two or more classes.
